# Early Ambulation to Prevent Delirium After Long-Time Head and Neck Cancer Surgery

**DOI:** 10.3389/fsurg.2022.880092

**Published:** 2022-04-07

**Authors:** Jeong Heon Kim, Yoon Se Lee, Yong Han Kim, Ki Ju Cho, Young Ho Jung, Seung-Ho Choi, Soon Yuhl Nam, Sang Yoon Kim

**Affiliations:** Department of Otolaryngology-Head and Neck Surgery, Asan Medical Center, University of Ulsan College of Medicine, Seoul, South Korea

**Keywords:** delirium, ambulation, head and neck cancer, postoperative care, survival

## Abstract

**Objective:**

Postoperative delirium is known to have various adverse effects on head and neck surgery patients. This study was designed to identify possible risk factors of delirium following long periods of head and neck cancer surgery and to help prevent postoperative delirium.

**Methods:**

We enrolled 197 patients who underwent long-time (>6 h) head and neck surgery at the Asan Medical Center from January 2017 to December 2018 in this study. Clinical covariates that may be associated with delirium were analyzed retrospectively using univariate and multivariate analyses.

**Results:**

Delirium occurred in 18 patients (9.1%). Within the first 7 days, 16 patients (88.9%) experienced delirium. Upon univariate analysis, delirium was associated with old age (≥75, *p* = 0.001), past neurological history (*p* = 0.019), time to ambulation (*p* = 0.014), and postoperative hospital day (*p* = 0.048). In multivariate analysis, old age (≥75, odds ratios (OR) 6.16, CI 2.00–19.00, *p* = 0.002), time to ambulation (OR 1.04, CI 1.01–1.07, *p* = 0.017), and past neurological history (OR 5.26, CI 1.09–25.37, *p* = 0.039) were significant risk factors associated with postoperative delirium.

**Conclusions:**

Older patients or patients with neurologic history must be attended with care, especially early after surgery. Encouraging early ambulation might lower the incidence of postoperative delirium and, subsequently, reduce adverse effects. This result could benefit patients by helping them avoid undesirable outcomes.

## Introduction

Postoperative delirium is one of the many complications that may arise in patients following extensive surgical interventions (1). It is defined as a change in mental status accompanied with a decline in consciousness and cognitive function (2, 3). The overall incidence of postoperative delirium in head and neck surgery inpatients is approximately 11%–26% (1, 4–10), and according to studies from various fields of medicine, this figure might differ with the different types of surgical interventions. In cardiothoracic surgical patients, the reported incidence of postoperative delirium is approximately 11.4%–55% (11), while in orthopedic surgical patients, it ranges from 3%–25% (12). In combined surgical and trauma ICU patients, the incidence of postoperative delirium is reported to be as high as approximately 70% (13). Such a condition usually develops over a short period of time and mostly exhibits rapid recovery. However, it is of concern to many health care providers due to its proximity with a number of unfavorable outcomes like prolonged hospital stay, which might lead to an increase in medical bills, higher morbidity rates, and higher mortality rates (14). Patients with postoperative delirium tend to exhibit a higher incidence of cognitive impairment (e.g., memory and concentration problems, sleep disturbances, etc.), and an increased risk of readmission (15). It is also known to have certain links with adverse events, such as pneumonia and death during hospital stay, though in the case of pneumonia, the exact causality is not well understood (16, 17). The factors leading to postoperative delirium are yet to be known due to its complex pathophysiology; however, there are a number of established risk factors that seemingly contribute to the development of this particular postoperative complication (6). Previous studies found that old age, the male gender, surgery duration, blood transfusion, free-flap reconstruction, and neck dissection were associated with postoperative delirium in head & neck cancer surgery patients (2). Advanced nodal disease was also significantly associated with postoperative delirium in free-flap reconstruction patients (18). Excessive hemorrhage, longer ICU stay, lower preoperative albumin and hemoglobin levels, and higher postoperative CRP levels were also risk factors for postoperative delirium in oral cancer surgery patients (1). Among these clinical variables, prolonged surgery, including free-flap reconstruction, extensive neck dissection requiring transfusion, and subsequent nutritional imbalance, are thought to be major predictive factors of postoperative delirium (1, 2).

Delirium is managed mainly through supportive care and pharmacologic treatment. Providing patients with repeated orientation to their surroundings, uninterrupted nighttime sleep, and visual/hearing aids to promote environmental interaction are well known protocols. The mainstream of pharmacologic management for postoperative delirium is haloperidol, which can be administered either orally, intramuscularly, or intravenously (19). Nevertheless, much time is required for some of the patients to recover from such conditions and to return to their daily lives (17). Therefore, early and adequate intervention for delirious patients would improve treatment outcome, and risk stratification of delirium in patients who underwent prolonged head and neck surgery might help patients receive such timely management, subsequently leading to an optimized treatment outcome.

This study aimed to further establish possible risk factors for postoperative delirium in patients who underwent extensive head & neck surgery and to analyze the impact of postoperative delirium on patient outcome.

## Methods

### Patients

A total of 275 patients who underwent prolonged (≥6 h) head and neck surgery at the Asan Medical Center (AMC) from January 2017 to December 2018 were enrolled in this study. Average follow-up period was 23.7 ± 14.2 months, and among these patients, otology and rhinology patients, patients with noncancerous conditions, and those under the age of 18 at the time of surgery were excluded from our list, leaving us with 197 patients. Surgery duration was measured based on the duration of the anesthesia. Data were reviewed retrospectively according to electronic medical records currently stored in the database of the AMC. This study was approved by the institutional review board (IRB No: 2021-1289) and the requirement for patients’ informed consent was waived.

Episodes of postoperative delirium were diagnosed according to the Diagnostic and Statistical Manual of Mental Disorders, 5th edition (DSM-V), which includes the following conditions; a) Disturbance in attention and awareness (reduced orientation), b) Develops over a short period of time (ranging from hours to days), fluctuates in severity during the course of day, c) Disturbance in cognition (e.g., memory, orientation, language, perception, etc.), d) Not explained by a preexisting neurocognitive disorder, e) Disturbance is caused by medical conditions or substance use (20). All patients diagnosed with delirium in our study met the above criteria and the diagnoses were reviewed by a certified psychiatrist.

### Variables

Preoperative variables included age, medical history (DM, HTN, psychiatric history, etc.), tobacco and alcohol consumption, performance status at admission, cohabitation, preoperative lab tests (albumin, hemoglobin, CRP, BMI), primary cancer site, and cancer staging. Cancer staging was evaluated based on the American Joint Committee on Cancer Staging Manual (8th edition) (21). Intraoperative variables included free-flap reconstruction, tracheostomy, and surgery duration. Postoperative variables included postoperative lab tests (CRP, hemoglobin), duration of ICU care, initiation of postoperative ambulation, postoperative complications (pneumonia, revision operation), and duration of postoperative hospital admission.

### Statistics

Statistical analyses were performed with SPSS 22.0 (SPSS Inc, Chicago, IL, USA). The threshold for statistical significance was set at *P* < 0.05. As for univariates, relevance with the occurrence of postoperative delirium were analyzed using the Mann–Whitney U-test and the independent Student’s t-test for non-normally distributed and normally distributed continuous variables, respectively. Categorical variables were analyzed using Pearson’s Chi square test. Statistically relevant univariates were further evaluated with multivariate analysis using the binomial logistic regression model. The regression model significance was reported as odds ratios (ORs) with 95% confidence intervals. Survival analyses were graphically visualized with Kaplan–Meier curves and compared based on the Mantel–Cox log rank test.

## Results

**Figure 1:**
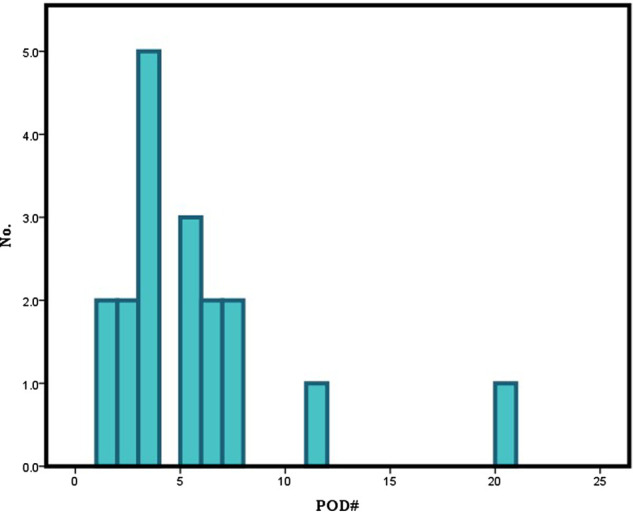
Onset of postoperative delirium in the 18 patients of the delirium group.

The demographic and clinical characteristics of the patients are summarized in Table 1. The mean age of the patients included in this study was 60.0 ± 13.3 years. Among these patients, 142 were male (72.1%), and 55 were female (27.9%). Of the enrolled 197 patients, 18 patients (male; 13, female; 5) experienced postoperative delirium. Thirteen of the 142 male patients and 5 of the 55 female patients developed postoperative delirium, showing no statistical significance (*p* = 0.989). Although age itself was not a significant variable (*p* = 0.082), dividing the patients by the age of 75 showed strong significance (*p* = 0.001). A majority of the patients (*n* = 16) usually presented delirious symptoms within the first postoperative week (POD 5.2 ± 4.5) (Figure 1). The enrolled patients were divided according to the location of primary malignancy; oral cavity (*n* = 102), oropharynx (*n* = 17), hypopharynx (*n* = 16), and larynx (*n* = 26). The rest comprised of patients with major salivary gland, thyroid, and skin cancer (*n* = 36). Anatomical site was not significantly associated with delirium.

**Table 1:** Characteristics and univariate analysis of preoperative factors related to delirium.

Variables	Delirium		*p*-value
	Yes, *n* (%)	No, *n* (%)	
Demographics			
Sample size	18 (9.1)	179 (90.9)	
Gender			0.989^a^
Male	13 (72.2%)	129 (72.1%)	
Female	5 (27.8%)	50 (27.9%)	
Age (y, mean ± SD)	65.2 ± 11.9	59.5 ± 13.3	0.082^b^
Age ≥75	7 (38.9%)	27 (15.1%)	0.001^a^
Medical history			
DM	3 (16.7%)	22 (12.3%)	0.595^a^
Hypertension	6 (33.3%)	58 (32.4%)	0.936^a^
Psychiatry	1 (5.6%)	1 (0.6%)	0.175^a^
Neurology	3 (16.7%)	7 (3.9%)	0.019^a^
Others	5 27.8%)	89 (49.7%)	0.076^a^
Smoker	9 (50.0%)	95 (53.1%)	0.803^a^
P-Y	31.1 ± 60.0	16.1 ± 22.7	0.664^c^
Alcohol	10 (55.6%)	104 (58.1%)	0.835^a^
Performance status			0.068^a^
Walking	16 (88.9%)	175 (97.8%)	
Sitting	1 (5.6%)	3 (1.7%)	
Lying	1 (5.6%)	1 (0.6%)	
Cohabitant			0.569^a^
Spouse	15 (83.3%)	117 (65.4%)	
Offspring	3 (16.7%)	36 (20.1%)	
Parents	0 (0.0%)	12 (6.7%)	
Siblings	0 (0.0%)	5 (2.8%)	
Others	0 (0.0%)	5 (2.8%)	
None	0 (0.0%)	4 (2.2%)	
Preoperative health status			
Albumin	3.6 ± 0.6	3.8 ± 0.4	0.250^c^
Body mass index	21.7 ± 3.3	23.1 ± 3.9	0.173^c^
Primary site			0.422^a^
Oral cavity	10 (55.6%)	92 (51.4%)	
Oropharynx	3 (16.7%)	14 (7.8%)	
Hypopharynx	0 (0.0%)	16 (8.9%)	
Larynx	3 (16.7%)	23 (12.8%)	
Others	2 (11.1%)	34 (19.0%)	

*Neurological medical history includes; stroke, brain tumor, Parkinson’s disease, etc.*

a *Pearson’s Chi square test.*

b *Independent t-test.*

c *Mann-Whitney U test.*

Among preoperative clinical covariates, preexisting medical history did not have a significant impact on the development of postoperative delirium, with the exception of neurological pathology (*p* = 0.019, Table 1). Neurologic medical history included conditions, such as stroke, brain tumor, Parkinson’s disease, etc. Smoking and alcohol consumption, performance status, type of cohabitant, and nodal stage seemed to have no significant effect on postoperative delirium.

Next, we explored the perioperative and postoperative clinical variates associated with delirium (Table 2). Perioperative lab findings such as albumin, BMI, CRP, and hemoglobin did not differ significantly between patients with and without postoperative delirium. The performance of free-flap reconstruction and tracheostomy did not differ significantly between the two groups.

**Table 2:** Univariate analysis of operative and postoperative findings related to delirium.

Variables	Delirium		*p*-value
	Yes, *n* (%)	No, *n* (%)	
Perioperative-OP lab			
Pre CRP	0.71 ± 0.96	1.07 ± 2.37	0.312^c^
Post CRP	12.92 ± 7.52	10.71 ± 6.82	0.195^c^
PreHb	13.2 ± 1.8	13.3 ± 1.8	0.924^c^
Post Hb	10.7 ± 2.0	10.7 ± 1.7	0.946^c^
Hemoglobin difference	2.6 ± 1.8	2.7 ± 1.5	0.656^c^
Free flap reconstruction	14 (77.8%)	113 (63.1%)	0.216^a^
Anterolateral thigh	6 (33.3%)	42 (23.5%)	
Radial forearm	8 (44.4%)	58 (32.4%)	
Others	0 (0.0%)	13 (7.3%)	
Tracheostomy	15 (83.3%)	121 (67.6%)	0.169^a^
Operation time (min)	642.4 ± 154.0	613.2 ± 162.0	0.430^c^
Intensive care unit (ICU)	14 (78%)	97 (49%)	0.054^a^
ICU days	1.1 ± 1.3	0.7 ± 1.7	0.065^c^
Ambulation (POD#)	19.1 ± 25.9	8.8 ± 10.6	0.014^c^
Pneumonia	5 (28%)	22 (12%)	0.069^a^
Post OP HD#	28.5 ± 26.0	23.0 ± 41.2	0.048^c^
pT stage			0.450^a^
T0, T1, T2	2 (11.1%)	51 (30.9%)	
T3, T4	16 (88.9%)	114 (69.1%)	
pN stage			0.397^a^
N0, N1	14 (77.8%)	102 (61.8%)	
N2, N3	4 (22.2%)	63 (38.2%)	

*Neurological medical history includes; stroke, brain tumor, Parkinson’s disease, etc.*

*POD# means number of Post-Operative Days, HD# means number of Hospital Days.*

a *Pearson’s Chi square test.*

b *Independent t-test.*

c *Mann-Whitney U test.*

The mean surgery time in patients with and without delirium was 642.4 ± 154.0 min and 613.2 ± 162.0 min, respectively, with the difference not being statistically significant (*p* = 0.130). The mean duration of ICU care and postoperative pneumonia showed marginal differences between the two groups (*p* = 0.065 and 0.069, respectively).

**Figure 2:**
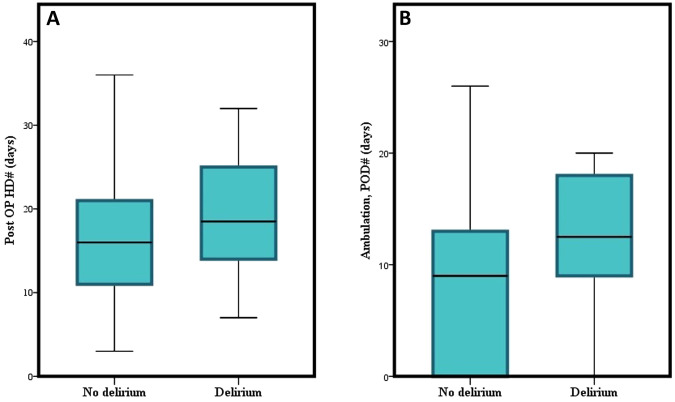
Graphical depiction of the difference of (**A**) postoperative hospital days, and (**B**) initiation of ambulation between delirium and non-delirium groups.

The initiation period of ambulation after surgery was 19.1 ± 25.9 postoperative days in the delirium group and 8.8 ± 10.6 days in the non-delirium group (*p* = 0.014). The duration of postoperative hospital stay also differed significantly between the two groups (28.5 ± 26.0 and 23.0 ± 41.2 days, *p* = 0.048) (Figure 2).

In multivariate analysis, neurologic history (OR = 5.26, 95% CI 1.09–25.37, *p* = 0.039), time to ambulation (OR = 1.04, 95% CI 1.01–1.07, *p* = 0.017), and old age (≥75) (OR = 6.16, 95% CI 2.00–19.00, *p* = 0.002) were significantly associated with postoperative delirium (Table 3).

**Table 3:** Multivariate analysis of factors predictive of delirium.

Variables	Odds ratio	95% CI		*p*-value
		Lower	Upper	
NR history	5.26	1.09	25.37	0.039*
Ambulation (POD#)	1.04	1.01	1.07	0.017*
Post OP HD#	1.00	0.98	1.02	0.941
Age ≥75	6.16	2.00	19.00	0.002*

*POD# means number of Post-Operative Days, HD# means number of Hospital Days.*

**Figure 3:**
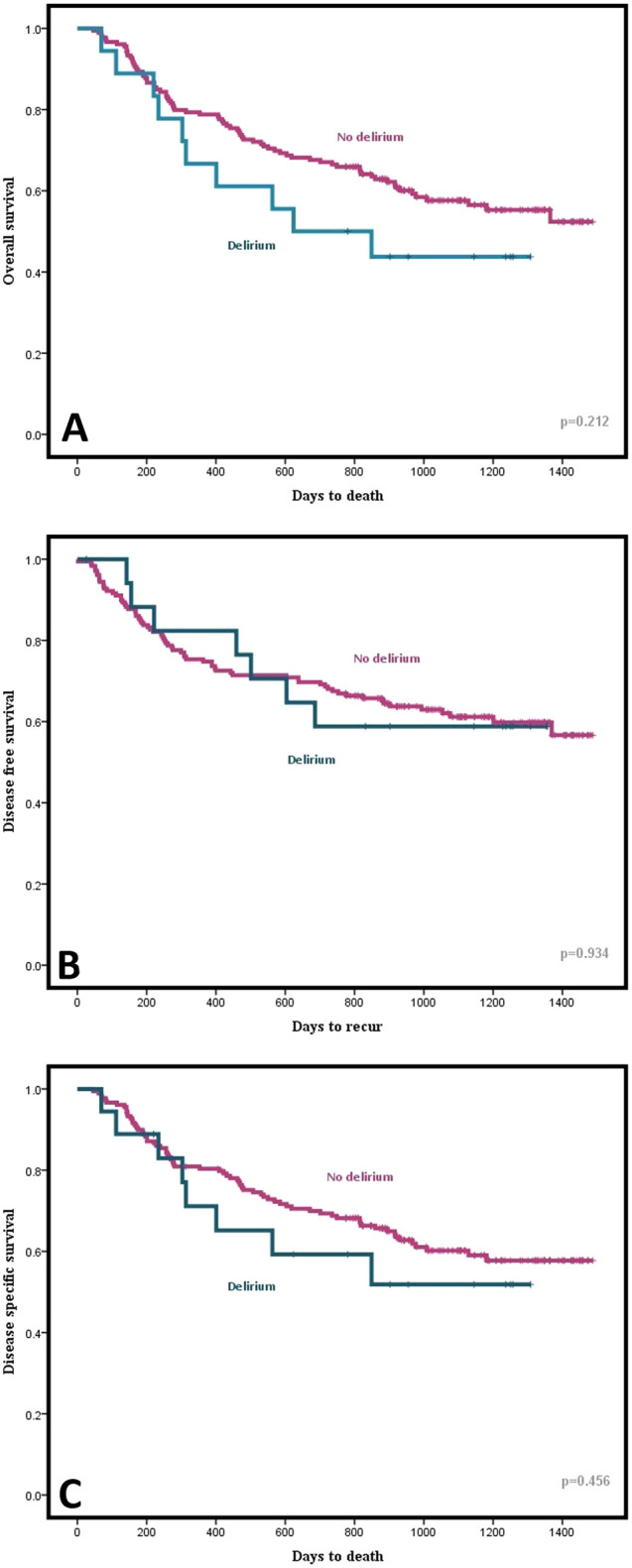
Graphical comparison of (**A**) Overall Survival (OS), (**B**) Disease Free Survival (DFS), and (**C**) Disease Specific Survival (DSS) between delirium and non-delirium groups using Kaplan-Meier curve.

Treatment outcomes, including overall survival (OS), disease-specific survival (DSS), and disease-free survival (DFS) were evaluated. In the Kaplan–Meier curves, the survival differences between the delirium group and the non-delirium group were analyzed with the Mantel–Cox log rank test. All forms of survival showed discrepancy between the two groups to some extent graphically; however, statistically, OS (*p* = 0.212), DSS (*p* = 0.456), and DFS (*p* = 0.934) all indicated no significant differences (Figure 3).

## Discussion

Delirium is a condition that is relatively well understood in adults (22). Previous studies stated that a longer surgery duration, specifically 6 or 10 h, significantly predisposed patients to postoperative delirium (5, 9). Therefore, to eliminate time as a confounding factor, we have targeted patients who went through extensive surgery in which anesthesia lasted >6 h. As a result, surgery duration had no significant effect on postoperative delirium in this study. In this study, preexisting neurological morbidity, old age, and time required for ambulation after surgery increased the risk of delirium after surgical interventions that lasted >6 h.

There have been a few risk factors for postoperative delirium. The incidence of postoperative delirium varies among different types of surgical interventions. Esophagectomy (16.4%) (23), oral surgery (15.4%) (1), and head and neck flap reconstruction (10.9%) (18) were predisposing factors. The overall incidence of postoperative delirium in this study was 9.14% (18 out of 197), which came close to the figures above, and does not deviate much from the findings of previous studies in which the incidence in head and neck surgery was 11%–26% (1, 4–10). Considering that other reports included all patients irrespective of operation time, our reports that include only the surgical interventions that last >6 h suggested a somewhat lower incidence of delirium. Both diagnostic criteria and formal reports from psychiatrists were likely to contribute to the incidence being lower than is seen previous reports.

Most patients began showing symptoms of delirium within the first 7 postoperative days (16 out of 18 patients, 88.9%). Such a finding is consistent with those of previous studies. Hasegawa et al. reported that most cases of delirium after oral cancer surgery (86.2%) happened between the 1st and 5th postoperative days (1). Takeuchi et al. reported that a majority of patients developed delirium by the 7th postoperative day (24). This is associated with nutritional or electrolyte imbalance, as well as with environmental differences. Careful evaluation would be necessary to check the occurrence of delirium for 1 week after surgery.

In spite of several attempts to identify the risk factors for delirium after surgery, no consensus has been made in setting a single model for predicting postoperative delirium (10, 25). Of the diverse factors mentioned above that are alleged to contribute to the development of postoperative delirium, old age, blood loss, longer surgery duration were widely acknowledged to have a significant impact (1, 2, 18). In our study, patients aged ≥75 had a significantly higher rate of postoperative delirium (≥75; 38.9%, <75; 15.1%). Marcantonio et al. introduced a model including the age of 70 years as a predictive factor, while Hasegawa et al. reported that the age of ≥75 years was a significant predisposing factor for postoperative delirium, which corresponds to what we found in the present study (1, 26). Blood loss was also one of the postoperative factors that was widely embraced as an important factor in predicting postoperative delirium (6, 9, 27–30). In the present study, we estimated intraoperative blood loss based on the difference in hemoglobin level before and after surgery. As hemoglobin level decline 2 days after surgery best reflects the degree of intraoperative hemorrhage, a comparison was made between preoperative hemoglobin levels and postoperative day 2 hemoglobin levels (31). However, unlike previous studies, intraoperative blood loss was not a major predisposing factor to delirium in this study. Other types of surgery, such as esophagectomy and cardiac surgery, would require transfusion more frequently than head and neck surgery due to less manipulation of major vessels. Compared to these studies, the number of operations requiring transfusion was not enough to evaluate the statistical significance.

The statistical relevance among ambulation and postoperative delirium was reviewed several times in previous studies, and time to ambulation was a notable factor, especially in patients who underwent orthopedic surgery. However, not much research has been conducted to unearth the significance of ambulation in patients who underwent head and neck surgery. Kamel et al. revealed that time to ambulation in hip fracture surgery patients was a significant predisposing factor to postoperative delirium (32). Takahashi et al. also established a significant relationship between time to ambulation and postoperative delirium in reconstructive surgery for oral cancer (33). In the present study, delayed onset of ambulation in patients turned out to be a statistically significant predisposing factor for postoperative delirium. This is compatible with the results of other studies mentioned above. Among the number of factors relevant to postoperative delirium, the onset of ambulation may be a major modifiable factor in postoperative care for head and neck cancer surgery patients. Therefore, encouraging patients to initiate early ambulation (if not contraindicated) seems to be a vital strategy for enhancing favorable patient outcomes.

Postoperative delirium is associated with higher morbidity and mortality, prolonged hospital stays, and higher costs. However, there have been some conflicting results concerning the differences in mortality rates between studies. Aaron et al. reported that the survival rate after esophagectomy did not differ significantly between groups with and without delirium. Zhang et al., on the other hand, revealed that those who developed postoperative delirium showed a lower OS rate (34). In the present study, we analyzed the prognosis of patients in terms of OS, DSS, and DFS, none of which yielded any statistical significance. Our short follow-up period might account for such results; however, as we can observe some graphical distinction between the two groups in the Kaplan–Meier curves, further research might be necessary to define the impact of delirium on patients’ prognosis. Although multivariate analysis excluded the length of postoperative hospital day from being associated with postoperative delirium, has still shown a significant difference between groups with or without delirium through univariate analysis. A number of earlier studies have similarly associated postoperative delirium with extensive hospital day (1, 9). Hence, particular care might be in need to prevent the onset of postoperative delirium so as to reduce hospitalization and medical costs.

This retrospective study has some limitations in data collection. Analgesics and sedatives, which might affect the occurrence of delirium, were not included in clinical factors. Given that the anesthetic effect in cardiac surgery was significantly associated with postoperative delirium, medication-induced delirium should not be overlooked (35). Furthermore, as delirium can be provoked by various factors including environment, conducting this study in a single tertiary hospital may also be a limitation to our results (36). Different clinical setting would be another predisposing factor of postoperative delirium. Multicenter and comparative study would resolve these limitations in the future study. In this study, we revealed that early ambulation, which was not usually described as a risk factor of delirium in head and neck cancer patients, may help to decrease the incidence of delirium. It would be difficult to define the exact cut-off value to avoid postoperative delirium because of a limited number of patients enrolled. Instead, this result that the earlier ambulation, the less postoperative delirium occurs will guide to shorten the period of bed rest, which is, however, variable between patients.

## Conclusion

Our findings suggest that old age (≥75), time to ambulation after surgery, and neurological medical history are significant risk factors for postoperative delirium. Ambulation period is the only factor that can be adjusted by physicians to prevent postoperative delirium. Early ambulation must be encouraged in patients with the abovementioned risk factors to prevent postoperative delirium and prolonged hospitalization. The efficiency of early ambulation in preventing postoperative delirium would be necessary to elucidate this result in future studies.

## Data Availability

The original contributions presented in the study are included in the article/supplementary material, further inquiries can be directed to the corresponding author's.
